# Extending Robot Therapy for Children with Autism Using Mobile and Web Application

**DOI:** 10.3390/s22165965

**Published:** 2022-08-09

**Authors:** Bojan Ilijoski, Nevena Ackovska, Tatjana Zorcec, Zaneta Popeska

**Affiliations:** 1 Faculty of Computer Science and Engineering, Ss. Cyril and Methodius University, 1000 Skopje, North Macedonia; 2 University Children’s Hospital, Medical Faculty, Ss. Cyril and Methodius University, 1000 Skopje, North Macedonia

**Keywords:** autism, robot, mobile application, web application, human–computer interaction, human–robot interaction, autism treatment

## Abstract

Robot treatments for children with autism have proven to be successful and effective. However, the resources needed for the treatments do not always meet the needs of the children. We overcame the lack of equipment and staff by extending the concept of robot therapy using a web and mobile application. This application enables greater availability and personification of the therapy itself. Its use in the majority of respondents contributes to improving their condition. This approach increases the flexibility of the therapy itself and makes it more accessible, enabling the patients to progress more rapidly. Although the robotic treatment presented in this paper is specific to children with autism, this approach can be generalized and applied to other areas where there are similar types of therapies.

## 1. Introduction

One of the most important features of human existence is mutual support and help. It is especially important when it is aimed at people who face difficulties in their lives different from those of ordinary people. It is, therefore, extremely important to understand their needs and to help them cope with every day challenges and function more easily in life. Autism is a disorder that affects not only the life and work of the person with the diagnosis, but also all the people in their surroundings. What characterizes autism is persistent deficiencies in the ability to initiate and maintain reciprocal social interaction and communication, as well as a range of other limitations. These generally include significant repetitive behaviors, interests, or activities that are atypical and/or excessive for the individual, age, or sociocultural context. The disorder usually begins in the developmental period, typically in early childhood, but symptoms may manifest and be detected later, when social demands exceed the individual’s limited capacity. The shortcomings are so serious that they can cause damage in personal, family, social, educational, and professional areas or in other important areas of functioning. Most often, the characteristics can be seen in all the principles of the functioning of the individual, although they may vary depending on the context [1]. What is particularly worrying is the fact that the number of diagnosed cases worldwide is growing: according to the World Health Organization (WHO), one in 160 children has autism spectrum disorder (ASD) [2], or according to [3], we have a median prevalence of 100/10,000. If we take a look at these numbers from the past, we can conclude that the number of children with autism has increased by more than three times in the last 20 years [4]. With the growth of the population with autism, the need for their appropriate treatment and care grows.

Because it is not simple to increase the number of resources needed in a short period of time, new approaches and innovations are needed to support and assist the process. Resources primarily refer to specialized professionals for interventions for people with autism whose number cannot increase rapidly and requires a long-term plan and time for appropriate training and education of this staff. What can be applied as a solution is to modernize the existing methods and enable them to be used without the presence of a child psychiatrist, psychologist, pediatric neurologist, or developmental pediatrician. This will allow the child to be on therapy on a weekly basis for a longer time without the burden on health care workers or in situations where additional therapies are not possible. However, this process is not easy and needs serious research into which aspects can be helped and in what way. This is especially true for digitization, which has proven to be extremely successful in many other areas, as well as a tool to help and support people with autism. Digitizing processes can make them more accessible to users and, in some ways, relieve them and assist doctors in their treatment of people with autism.

One of the unconventional treatments that gives promising results is the inclusion of a robot in the therapies. Several different types of robots are used in these treatments, among which, the most common are NAO, Kaspar, ZECA, ZENO, Mina, Probo, Troy, Robota, Keepon, etc. [5,6,7,8]. Robots in these treatments can be used for a wider range of activities and can play a different role; furthermore, their purpose can be changed multiple times during one session. These studies show that robots are effective in improving the learning, social skills, and communication of children with autism [9]. However, these therapies are still in their infancy, and in the future, in order to make them better and to show their meaning in a better light, several guidelines should be followed such as the inclusion of guidelines as in clinical research that will determine evidence-based practice [10]. Furthermore, the small samples on which these studies are carried out, as well as the absence of control groups are a common practice in this type of research [11], as well as the fact that it is not clear enough whether the social–emotional dimension of human–human interaction can be fulfilled through robots [12].

Nevertheless, their specific characteristics help children to behave more freely and feel safe in their environment, which usually encourages attempts at communication and sharing and could be used as a facilitator in autism treatment [13,14]. Because robots are less complex than humans, it is easier for children to follow instructions, as well as to interact with the robot without having to use complex verbal and non-verbal structures in communication, but it can also trigger communication between children [15,16]. Usually, this type of treatment is implemented as a game that allows children to learn more easily and practice new skills in a confidential and safe environment. Robots can be used in the very process of diagnosing autism, but also as mediators, personal therapists, and instigators of sensory, cognitive, social, emotional, and motor events [17]. Therefore, the inclusion of human–robot interaction in therapies for children with autism can play a significant role and stimulate children’s interest in certain activities, but also help parents and practitioners. However, there are also problems in this approach, and one of the problems, faced by those involved in this type of therapy is the lack of hardware, robots, and therapists trained to work with them [18,19]. These limitations make this type of therapy difficult to access for most children with autism, but also, those who have the opportunity to be part of this therapy are limited in the number of sessions they can have, which is a problem for children who respond positively to this approach because there is no room to increase the intensity of the therapy itself.

In order to improve this and give the children the opportunity to be involved in such sessions for longer and more intensely, we introduce a hybrid model, a combination between sessions with a robot and a mobile and web application [20]. The purpose of the application is to have the best possible reflection of the therapeutic sessions, which would overcome this problem of a lack of resources. Applications themselves are another segment that generally positively affects children with autism mostly in terms of social interaction, i.e., they engage in more verbal exchange with the robot and are more physically involved in the interaction [21]. Research has shown that mobile and web applications have many advantages such as the fact that they are intuitive and easy to use, easily adapt to the needs of people with autism, are visually appealing, and provide consistency [22], and they have a positive affect on children with autism in terms of learning, language, social interaction, and skills [23,24,25]. They are also portable and can be used in a variety of conditions, which is of particular importance for our research. Another advantage is that they are easily accessible and will allow us to bridge the gap we have in terms of hardware unavailability. Additionally, applications and IT tools offer huge opportunities in terms of easy modification and customization of content to users, as well as easy tracking of how they use the application and their progress. This allows them to be customized for the user, and this can be from the application itself or remotely. In this way, the treatments are personalized, and in a very easy way with very little effort, each child can obtain an application with content designed only for her/him, even without the need to be in physical contact with the therapist or the person in charge of it.

Needless to say, developing applications for children with autism also has its challenges such as how to incorporate theory into technology, that it is difficult to measure utility and isolate improvements, as well as how to obtain adequate feedback from users, the small number of users, the development of the relationship with users, and ethical issues [26,27]. Because it is a very specific user group, in which users share some features, but also differ significantly from each other, it is really difficult to generalize the requirements and fit them into one application. In this paper, we describe several aspects of the new hybrid method for using a mobile and web application as a complement and extension to robot therapies. It will include a section on how the application itself was built, the analysis of questionnaires intended for parents and psychiatrists related to the application and the whole process of such kind of therapy, as well as the analysis of the data from the usage of the application itself. The first section gives an introduction to the problem. The second presents the approach used and the way the solution is implemented. The results are elaborated in the third section, and the last two section present the discussion about the solution and the conclusions of the research.

## 2. Subjects and Methods

### 2.1. Subjects

In this paper, we present results from 20 children, diagnosed with autism, aged 23 to 76 months (AVG = 47.5; SD = 14.04). All children were already receiving regular treatments and they attended robot therapy with the robot Kaspar. Furthermore, they used the Kaspar application in home settings. Therapies were received in Macedonia and Croatia.

### 2.2. Methods

**Figure 1:**
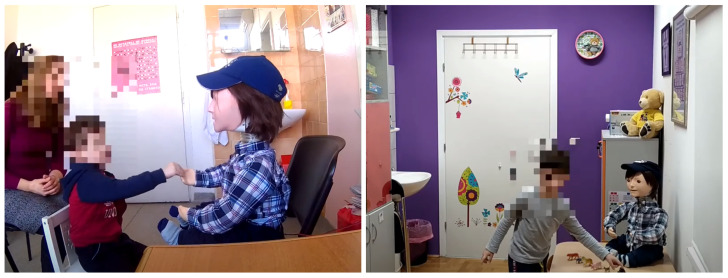
Therapeutic sessions with the Kaspar robot.

Kaspar is a humanoid-sized child robot with a simplified look that has the ability to move the head, arms, and torso, facial expressions, and speech (previously recorded) [28]. The robot also has several touch sensors on the head, body, arms, and legs and has the ability to hold objects such as a comb, toothbrush, spoon, etc. This allows for a wide range of activities that the robot can perform. The therapy is performed in such a way that Kaspar is placed in front of the child and is controlled by a pediatrician via a remote keyboard or computer (Wizard of Oz experiment) (Figure 1). In this way, an interaction is established between the child and the robot. The robot itself already has programmed exercises that include movement, facial expressions, and speech. The therapist leads the session by performing a variety of exercises. In this way, through a wide range of therapeutic and educational exercises and games, such as turn-taking, joint attention and collaborative games, cause and effect games, etc., the therapist can keep the child engaged and interacting with the robot. Exercises and games can be as varied as expressing emotions, various greetings, choreographed songs, daily activities, learning exercises, and other short conversations and phrases used in daily interaction. Pre-recorded speech allows these exercises to be easily adapted to different languages. The process itself may include additional items such as toys, cutlery, instruments, etc., which further enriches the whole process and enables the creation of an even wider range of exercises and games.

**Figure 2:**
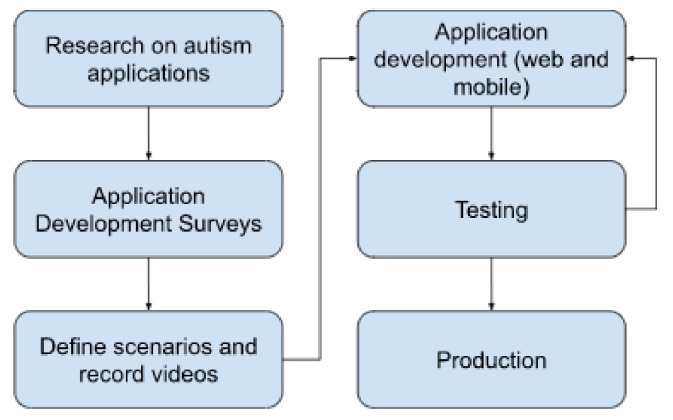
Phases of the development process.

A web and mobile application was developed as an extension and expansion of the robot therapy process for children with autism. The main idea was that the application aims to mimic the therapy process and enable it in domestic conditions to some extent. The process of gathering information for the application development involved children attending robot therapy, as well as their parents and the specialists conducting these therapies. The development process is explained in Figure 2.

Since we have a relatively small and specific group of children for which the application is intended, the first step was to research and gather experiences from other applications. This helped greatly in the user design part in order to cover some already known requirements for the user group that we targeted, i.e., children with autism. Although individuals with autism are very different from each other, there are generally some already established principles for designing applications for these individuals [29,30]. The first and basic principle is the simplicity of the user interface and the general design, and this is especially important because of the way these people perceive things [31]. The size of the screen and the elements placed on it should be made large enough for the users to be able to see them and take action, usually between 5% and 20% of the screen size [32]. Furthermore, through the size, we can define which elements of the screen should have more attention paid to them [33]. The number of images on the screen at a time should not be large and within certain limits that are defined by the size, i.e., the screen space and the size of the images themselves [34]. Similar to images, the sounds within the application should match the images, videos, or actions that users can take, but we have to be very careful with the use of sound and music because, sometimes, it can distract the child [35,36]. In terms of colors, what is recommended is the use of atypical colors and colors that are not intense because these people experience colors differently as a result of their increased sensitivity to sensory stimulation [37,38]. Given the fact that they have damaged or delayed language, we should be extremely careful with the usage of text in such applications, and it is usually recommended that the text be supported by images [39,40,41]. It is extremely important that the navigation be simple [42]. Although we were limited in terms of design, because the application had to rely heavily on the design of the elements of the Kaspar robot, through our experience in developing such applications [43], we tried to stick to these recommendations as much as possible.

**Figure 3:**
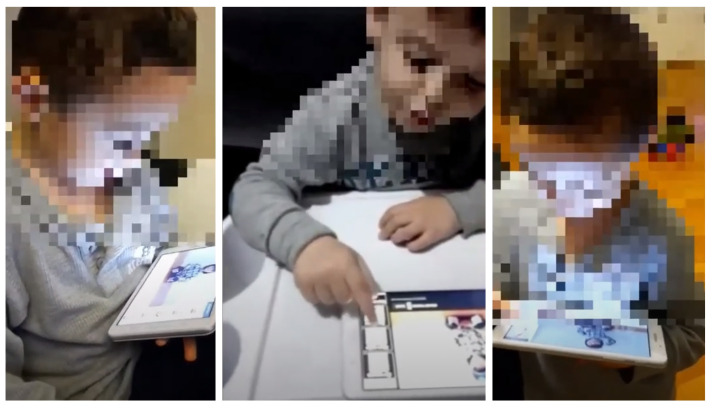
Children while using the application.

**Figure 4:**
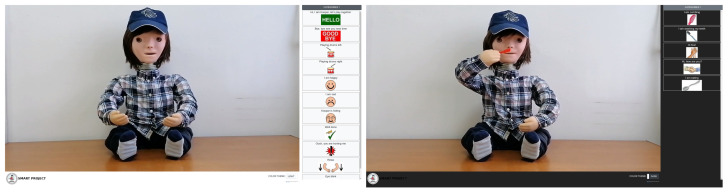
Main page web and mobile application (light and dark theme).

The other factor that also had a big impact on the application development was the answers we received from the parents of the children for whom this application was intended. For that purpose, a questionnaire was prepared, i.e., a survey was distributed to the parents before starting any design of the application. The purpose of the survey was to find out their needs and expectations from the application. With the information we summarized from the first two steps and under the influence of the already existing design of the robot, we were able to form initial sketches for the design of the application itself. A more detailed analysis of the surveys is given in the following section. The next stage involved defining the scenarios. In this case, the scenario is in a way mimicking the treatment exercise that the doctor performs along with the child and the robot. The idea was to determine the scenarios and auxiliary elements that would be used in the application. Therefore, as a part of this process, the most appropriate scenarios were selected, and they were divided into fivecategories of standard scenarios, songs, actions, food, animals, and body parts. The scenarios were chosen in such a way as to cover as wide a range of different things (actions, daily activities, exercises, songs). The next phase was the creation of the scenarios. One scenario represents one or more sequences of related actions performed by the robot Kaspar during the intervention [44]. In order to create as recognizable an environment for children as possible, we set the stage to be the usual environment that children have during the interventions. Kaspar was placed where he usually stands, and we made several videos for each of the scenarios from different angles, in an attempt to generally simulate the child’s view of Kaspar during the interventions. After completing the videos, the next step was to edit and improve them, as well as convert them to an appropriate format in order to maintain the quality, but to be small enough in memory so that the whole application can be easily placed on a mobile device and a website. One of the requirements we received when analyzing user requirements was for the videos to be of satisfactory quality and to be able to load quickly even on devices with lower performance. Therefore, this step was crucial for the further success of the application. The audio was also removed from the videos, so that we could put different speech in the same video in different languages, because according to the requirements we had in the project, the applications had to be delivered in three different languages (Macedonian, Croatian, and English). Additionally, this approach would provide a simple way to add new languages in the future, without the need to modify the videos. The next step was to edit the sound and add it to the videos in order to produce videos in different languages. The developed web and mobile application were installed on tablets, which were distributed to the children (Figure 3), and also, the application was placed online in the form of a website (Figure 4) [45].

In order to make the application easier and more attractive to the users, as well as to collect additional information on user behavior for further analysis, we implemented a logger that recorded every action that was performed within the application. This logger saved all the actions of the user, i.e., all clicks and taps in the application. This allowed us to perform an analysis of where and how much the users clicked, whether the clicks meant the release of a script or were empty, how often a script was played, and so on. The data were collected and stored in a file on the device itself. The parents of the children who used the application were informed about this way of collecting data and for what purpose it was performed, and the only way to access them was for them to voluntarily give us the log file.

## 3. Results

During and after the development of the application, in order to find out the requirements of the users, as well as their satisfaction using the application, four questionnaires were created that considered different aspects of the application: application usability questionnaire (before application development), application usability questionnaire (after application development), developmental evaluation questionnaire (before and after treatment), and usability questionnaire for the professionals. In the sequel, we will discuss the features of each of the questionnaires.

### 3.1. Application Usability Questionnaire (before Application Development)

This questionnaire was compiled and distributed to the parents of the children who attend treatments with the Kaspar robot, and the purpose of this questionnaire was to collect data about parents’ expectations from the application in terms of its appearance and performance. The questions selected for this survey refer to the elements that were considered important in the development of this type of application and the elements that are characteristic of this application. The survey consisted of 11 questions with answers in the form of a ranking scale from 1 to 5, where 1 corresponds to strongly disagree and 5 to strongly agree. Parents of 20 children participated in the survey, and the results are presented in Table 1.

**Figure 5:**
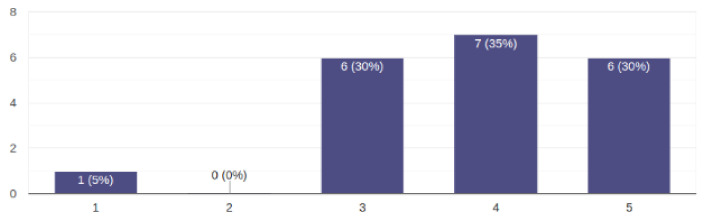
Answers regarding quality of the videos.

As can be seen from the results presented in Table 1, 9 out of 11 questions had an average grade of 5 or close to 5. This means that parents thought that easy use, navigation, use without prior instructions, easy handling of wrong actions, and the ability to quickly learn to use and have fun were of great importance. Regarding the quality of the videos according to the average answer, the parents thought that it was important, but not too important. The results on this issue are presented in Figure 5.

**Figure 6:**
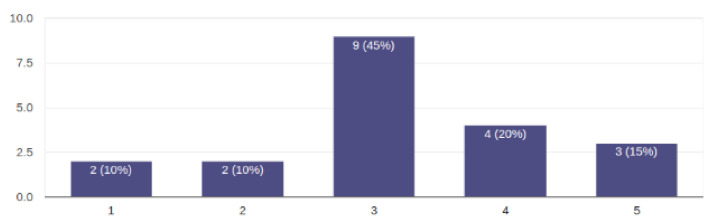
Answers regarding on-screen additional messages and announcements.

Regarding the question of whether additional messages and announcements could be disturbing for the child, the average answer was neutral, and the results for this question are presented in Figure 6.

The results of this survey were of great importance in the application design and development process. The application usage was reduced to an average of two steps (clicks, taps) to start the desired scenario, category selection and scenario selection, or if the screen was not large enough, scrolling through the scenarios and selection. Making mistakes was kept to a minimum. One of the possible mistakes is choosing the wrong scenario, but it can be corrected just by choosing a new scenario. In this case, the old scenario stops and the new one starts immediately. The images used for the buttons were the same as the special keyboards used to control the Kaspar robot, so most of the users were already familiar with them and it was easy to map which button refers to which scenario. In terms of video quality, although the requirements here were not so strict, we thought it was important that the video quality be high, and in this case, we chose the best ratio between video quality and size. We wanted the application to be able to be used without an Internet connection, so all the videos had to be packaged in the application itself. However, it was also necessary that the application be not too large and the videos could be loaded quickly. For this purpose, a compromise had to be found between the quality of the videos and their size. Regarding the last question about additional help messages, we decided not to implement any messages that would be distracting. The goal was to see if the application could be used effectively and if it was clear enough, so in one of the following versions, we could add explanations and help where needed. From the observations and comments, we received later during the use of the application, we realized that there was no need for such messages.

### 3.2. Application Usefulness Questionnaire (after Application Development)

This questionnaire was intended for the same group of parents, and it was distributed after a certain period of use of the application. The purpose of this questionnaire was to gather information on how the children are using the application, its usefulness, how it works, problems, etc. The questionnaire consisted of 36 questions, 3 of which refer to the manner and frequency of use and have predefined answers, and the remaining 33 generally refer to the experience with the application; the answers are presented through a list of possible answers from 1 to 5. Some of the questions are supported with the possibility of leaving comments, in order to obtain better information from the parents, i.e., comment on their response or opinion on the matter. According to the answers to the questions about the frequency and duration of the sessions, there were 14 users that used the application for 5–10 min and 4 users that used it 10–20 min, once or twice per week. Furthermore, there were 2 users that used it 5–10 min 3–5 times per week.

Fifty-five percent of the respondents answered that they used the application following the instructions and directions given by the therapist or more, while the rest answered that they did not use it following the instructions or followed them less than recommended. The answers to the other questions were offered as a scale from 1 to 5, and the number of answers with a grade per question are presented in Table 2.

In the first column of the table, the questions are shown, and in the other five columns, the number of answers with that grade for the respective question are presented. The cells in the table are colored according to their value in a gradation of white for 0 answers to green for 20 answers, in order to better visualize the values of the table itself. As can be seen from the results of 28 out of 33 questions, most of the answers were mostly positive or, on 27 questions, more than 50% of the respondents gave a mostly positive answer. As already mentioned, in the framework of this survey, space was left for so-called open questions, i.e., questions that can be answered in the form of free text. This allowed us to obtain more detailed information about the whole experience. Although such responses were not numerous, they contributed to a better picture of the problems faced by parents during use. One of the parents had commented that the child refused to use the application, was not interested, and asked to be excluded. A comment from another parent was that he personally did not see the point in this approach, that he did not want the robot, and that he was afraid of it, but that the child did not complain when using the application. There were also comments that referred directly to the system and the design itself, i.e., there was a complaint that, on one device, the system slowed down and there was a large distance between the images and the text. In the other comments, it was pointed out that the application was interesting for the child, it enabled them to practice and improve some skills, as well as that it facilitated the work of the parent in a way that he/she did not have to devise activities himself/herself. According to the results of this survey, it can be concluded that the majority of parents thought that the application was good quality, its operations were smooth, and it fully corresponded to its purpose and goal. A quarter of them believed that the additional use of the application supported the therapy itself and increased its effectiveness. The additional comments allowed us to be better acquainted with the opinions of the parents and pointed out errors and problems, which allowed us to remove them and improve the application itself. They even pointed out some things that were not the primary goal of the application itself, that is it can serve as a kind of guide for exercises that can be performed at home. It should also be noted that the interpretation of the results should be made with special caution, because it should be borne in mind that the answers are subjective and that the concept of the scale may vary between parents. Furthermore, the answers, i.e., the assessment, can be influenced by the expectations that the parents have of the child, the treatment, and the application. Therefore, the same assessments of the same questions from different parents may be different from reality. Even the same scores on different questions from the same parent can have different weights.

### 3.3. Developmental Evaluation

The developmental evaluation questionnaire was completed by the parents and generally refers to the behavior and characteristics of the child in several categories. This questionnaire was completed before and after the end of the treatment and served as an indicator of the changes that occurred in the child during the treatment. The questionnaire consisted of 75 activities or behaviors divided into 12 categories, and the answers refer to the frequency with which the child performed the activity. The answers were conceived of as a five-level scale and can be never (you have never seen a child behave this way), infrequent (the child rarely behaves this way), occasionally (the child occasionally behaves this way), often (the child behaves in this way regularly/many times in a short period of time), always (the child always behaves in this way), and not applicable (the question is not applicable given the behavior and skills of the child). The names of the categories, as well as the number of questions per category are given in Table 3.

As mentioned, the analysis of responses to the child’s behavior before and after treatment is extremely difficult to perform for several reasons. One of them is the way the questionnaire is designed. The offered answers are given in the form of ordinary values such as “never”, “rarely”, “occasionally”, “often”, and “always” and an additional choice, “not applicable”, to questions that are not applicable to the child’s behavior. Ordinary values are categorical values and can be sorted, but the distance between the values is unknown. In our case, we could not say that the distance between never and rarely is the same as the distance between rarely and occasionally or often and always, or we could not say that the distance between occasionally and always is twice the distance between never and rarely. In our case, distance would be the frequency of activities or features observed in the child. According to this, it is difficult to measure the progress that the child has before and after the treatment. Another factor that exclusively affects the data is the subjectivity of the parent who filled out the survey. It is difficult to determine how the parent maps between the frequency and one of the offered answers and what each of these answers means to him/her. That is, it is quite difficult to determine what the limit is, for example, with what frequency the child should repeat the action in order to characterize it as as rare or occasional. For different questions, this scale can answer different frequencies, depending on how the person who filled out the survey perceives the behavior of the child. Another factor to consider is comparing answers for different children. This is also extremely difficult to do, because as we mentioned, different parents perceive their children’s behavior differently. Therefore, it is possible that what for one parent is rare, for another is occasional or frequent, or that what for one parent it would mean a change in two steps from rare to frequent, the same behavior someone else perceives as occasional in frequent. Often, these ordinal values are mapped to integers, such as the Likert scale (Cohen), but it should be noted that, even in this case, the distance between 1 and 2 is not equal to the distance between 3 and 4. Knowing this, the analysis of these questionnaires should be performed very carefully. One way to analyze these types of questionnaires is by reporting the amount of change. Furthermore, analyzing the overall results may lead to the wrong conclusions, as the aggregate results may be positive, but many individuals may have reported a decrease in frequency, especially in our case, where the analysis per individual was extremely important.

**Figure 7:**
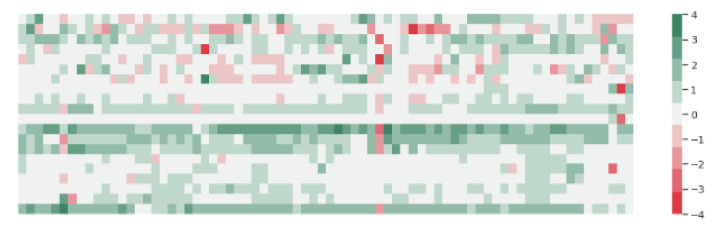
Color map of change. Red indicates a negative change; green indicates a positive change; white indicates no change. Each row is a questionnaire answered by a parent; each column is a question.

In order to somehow present the change in the answers to the questionnaire before and after the therapy, we recalled the difference in the change in the answers to each question for each child. To achieve this, each of the answers was mapped to an integer from 1 to 5 as follows: 1—never, 2—rarely, 3—occasionally, 4—often, 5—always, after which, we calculated the difference between the response after and before therapy. The results we obtained per question could range from −4 to 4. In this case, −4 would mean the maximum negative change in frequency, i.e., the answer was “always” before therapy and “never” after therapy, while 4 would cover the opposite case and represent the maximum positive change. The other values include smaller positive or negative changes, and 0 would mean no change in the answers. Figure 7 in the form of a color map table shows the change in the answers to the questionnaires, so that, in each row, one questionnaire is presented and each column is a question. The change is marked with a color scale, where green indicates a positive change, white indicates no change, and red a negative change. The magnitude of the change is indicated by the intensity of the color, green and red. What we can generally notice is that white and green are predominant, which would mean that there is no change or that there is a positive change. This overview gives us an idea of the trend, but questionnaire or query trends can also be detected. For example, it can be noticed that in the second questionnaire, there are significantly more red fields than the others, or that in the seventh question, on all but two questions, we have no change, or that in 11 and 19, green is predominant. We can also notice a trend per question, such as that for the 44th question, there are the most negative changes. A positive change does not mean that we have an improvement, nor a negative change that we have a worsening of the situation. What we measure is whether there is a change in frequency, and it depends on the question whether it means improvement or not of the child’s condition. For example, 44 reads “The vocalization of the child involves chattering with intonation as if he were speaking (as if the child were speaking his own speech)”, and in this case, reducing the frequency would mean that there is progress. This is the only issue where frequency reduction has a positive effect on its progress. For that reason, in the further analysis, the values for the changes in this issue were taken with the opposite sign.

In order to performed a better analysis and obtain a better picture of the changes per child, we created Table 4, which presents the questionnaire per patient in rows and the number of positive and negative changes in columns or that there was no change. The color of the cells is obtained with white to green, where white is the smallest value and green is the largest. This made it easier for us to detect trends visually. As from the previous picture, what can be noticed is that the trend is to have no changes or to have positive changes, i.e., if we look at the summary, only 8% of the questions have a negative change, 49% have no change, and 43% have a positive change. Looking at the questionnaire, 6 questions were dominated by positive changes, while 13 were dominated by the fact that there was no change, and in one, we have an equal number of questions that did not change and had a negative change. If we look only at the way the table is colored, we can see a generally positive trend or that there is no change. This gives us encouraging signals that the approach we used may encourage positive change, in some respects, in some of the children.

As already mentioned in the description of the questionnaire, the questions were divided into categories. In Table 5, we present the percentage of changes in the questions by category. Again, the color in the cells is represented by a scale from white (lowest value) to green (highest value). The trend by category is that there is no change or that there is a positive change, and in general, the percentages between these two items are similar. The categories, where we have more drastic deviations are vocalization and speech, coping mechanisms, and cause and effect, where questions that do not change prevail, and everyday skills, where answers that represent some improvement prevail.

### 3.4. Questionnaire for Professionals

This questionnaire was intended for professionals, i.e., therapists, and its purpose was to see their experience working with children, the robot, and the application. There were just five therapists involved in the process; therefore, there were five completed questionnaires. The questions in this section were of different types and were divided into several categories (Table 6).

Regarding the frequency of using the robot in therapies, three therapists answered that they used it 1 to 2 times a week, one 2 to 3 times a week, and one therapist daily. In terms of the duration of the sessions, one answer was 5–10 min, and four answers were 10–20 min. In the part about the reaction and the use of the hardware, i.e., the robot itself, the answers were on a scale of 1 to 5, where 1 means “I do not agree at all” and 5 means “I completely agree”. Furthermore, some of the questions had the opportunity for open answers, in order for the therapists to give their opinion on the matter. In terms of meeting the requirements of the therapist and the child, the assessment was somewhere in the middle to a slight inclination towards a positive opinion. The comments in this section were that it is a good help to acquire some of the skills and makes it easier to direct the child’s attention, as well as that some of the children responded better than to some conventional methods. In terms of usage, again, the answers were somewhere in the middle of the scale with a slight tendency towards a positive experience. In terms of learning speed issues, the robot’s fun elements and overall satisfaction prevailed. Regarding the questions related to the application, the therapists had a generally neutral attitude as to whether the application met their needs and a positive attitude that it met the needs of the child. The comments in this section were that the child responded well to the actions of the application and that it was easy to use, and there were suggestions for adding more scenarios to the application itself. The answers to the questions about the ease of use of the application (from the therapist and the child), the number of steps needed to take some action, the possibility of use without written instructions, and the fun elements in the application were mostly positive. The situation was the same as the answers to the questions related to the design of the application, the text, the visual organization, and the quality of the videos. The answers to the questions related to learning to work with the application and the capabilities of the system were also generally positive. Regarding the very concept of using Kaspar and the application, the answers were extremely positive. The therapists thought that, for some of the children, the hardware was more interesting, i.e., the Kaspar robot itself, because they could interact with it, while others were afraid of the robot, and for them, the application was a better option because it was easier to apply and allowed the continuation of therapy at home. Particularly positive comments were directed at the greetings and imitations of animals because the children managed to generalize them in real life. The general opinion was that the concept itself was a good auxiliary tool in the process of therapy and improvement and encouragement of the child’s skills through the use of high-tech devices.

### 3.5. Logs from the Use of the Application

**Figure 8:**
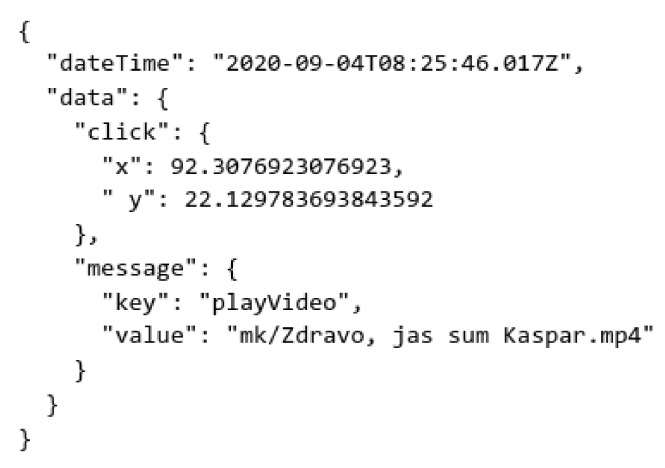
Example of one log.

As mentioned in the section on application development, a logger was built into the application in order to monitor the work of the users. In this attempt, activities from six devices were successfully collected. The logs were .json files of 6.9 KB to 1.4 MB and were taken directly from the device with parental approval. Every entry in those files was an event of the application; an event can be a click (touch) or launch of the application. For each event, the following information was stored: the time when it happened (dateTime), in which position it happened on the screen (data.click), and which action happened (data.massage) (Figure 8).

**Figure 9:**
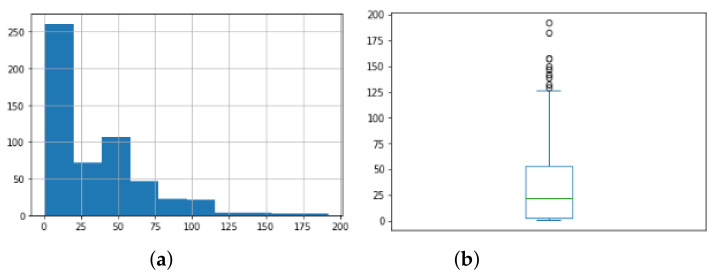
(**a**) Histogram for number of events. (**b**) Box plot for number of events.

For easier implementation of some of the analyses, the json format was restructured in tabular format, and attributes such as session name and duration of the event, i.e., time spent between two events, were added. The number of logs per file varied drastically, which was to be expected if we know that the application would be used at a different frequency by different users. For better analysis, we grouped the events into sessions. A session is a period of continuous use of the application. However, we noticed that if we took the duration of a session from running an application to the last event before re-running, we obtained sessions that lasted up to 14 days. This is because the application can be seen left running in the background. In order to make the sessions more relevant, we considered that the session was over if five minutes had passed without any activity, i.e., if no event was registered for five minutes. There was a total of 17,840 events and 370 application launches. Within a session, we can look at how many events there were and how long a session lasted. This would give us an idea of the length of the sessions and the activity of the users within the session. Figure 9a shows a combined histogram for the number of events per session of all users, and it can be seen that, in general, sessions tended to have between 0 and 100 events. This can be seen better in Figure 9b, where the box plot of the number of events per session is presented. The average number of events per session was 33, but it should be taken into account that we also had outliers and that 75% of the sessions had less than 53 events, which can generally be seen from their distribution shown in Figure 9.

Another thing that is interesting to observe is that a quarter of the sessions had three or fewer events, which would mean that the application was launched, but was not used at all or, more precisely, there are 107 sessions with one or two events.

**Figure 10:**
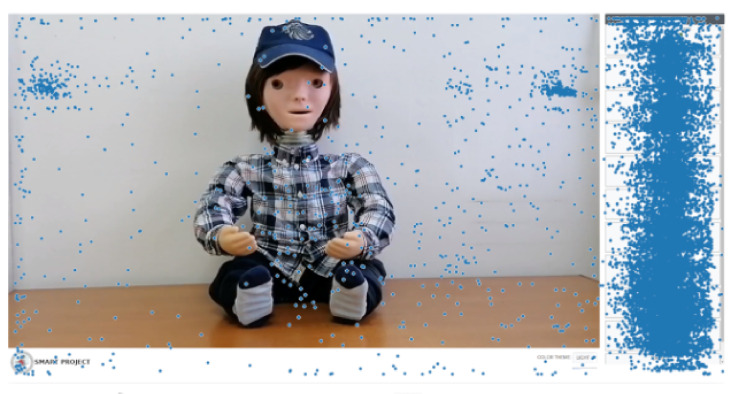
Positions of the all clicks/taps on the application.

Figure 10 shows all the clicks/taps of the application in the form of dots, and almost all of them were involved in the selection of scenarios; however, there were some that were on the videos, most likely by mistake or out of curiosity, which would happen if clicked there. Two more specific areas were many events separated in the upper left and right corner of the video; these were probably unintentional thumb touches while holding the device. We can also see touches on the edges of the screen probably for the same reason, accidentally while holding the device.

**Figure 11:**
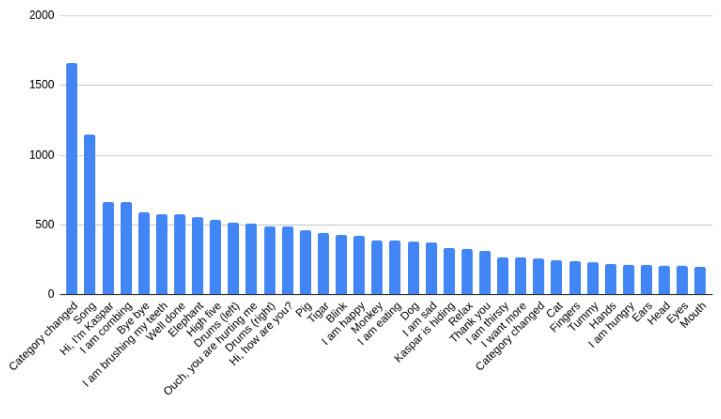
The most played scenarios.

We also counted the number of scenarios played at all sessions, and we noticed that the song “Clap your hands” was played by far the most, almost twice as much as the next one in the list, “Hello, I am Kaspar”. Then followed the scenarios “combing”, “bye bye”, “brush your teeth”, etc., as shown in Figure 11.

## 4. Discussion

The results of the questionnaires showed that the requirements and expectations of the users from the perspective of the appearance and use of the application were met and a way has been found to successfully incorporate them all into the application. The questionnaire regarding the use of the application indicates that it is easy to use, without irregularities, with excellent organization of the structure in it, and high-quality multimedia elements. Although the application was rated as fun and satisfying with interesting elements, about the results regarding the level of use of the application by children, we can say that the usefulness of the application is at a solid level, and there is potential to take it even higher. Regarding the effects of the application itself on the development of the children, the comments were generally positive, and the parents believed that the application can contribute to improving the condition of the children in many different aspects. Pediatricians also believed that this approach had a positive effect on children. Another benefit of using the application is the logs it collects. This allowed, with a later analysis, to see what were the most-used scenarios, when did the child select each scenario, how often the scenario changed, and so on. This enabled individual analysis of each of the children who used the application. Therefore, individual fine-tuning of the use of the application is possible. This would range from simple statistical analyses of counting certain events to more modern techniques of machine learning and pattern unlocking or time series clustering. This research also had some drawbacks. One of them is the small number of children included in the initial research. This is a brand new concept, so we have not been able to test it on a wider group. Another disadvantage that arises is the lack of a control group. In this case, all involved children were included in intervention that included the robot Kaspar in their activities and were additionally given a tablet with an application installed. Since the number of participants was small and we only tested the concept, there is no group that is available for robot therapy, but does not have access to the application. However, since ASD has individual manifestations in different participants, it is extremely difficult to make general conclusions, and the results should be observed individually. Here, it is necessary to take into account the fact that all the participants in the meantime attended other therapies, so any progress cannot be attributed to this approach alone. One of the challenges we faced was the COVID-19 pandemic. Therefore, for some time, the participants were not able to attend the robot therapy, and they were left with only the use of the application at home. This in a way gave additional importance to the application itself because it proved to be a potential solution in such an emergency situation. The newly created situation showed us an additional potential of the whole concept and presented different views of the process itself. As we have already mentioned, one of the improvements that can be made is to increase the number of participants in this type of therapy and to increase the period of the process itself. This would contribute to more data and the ability to analyze them, as well as to build models and find patterns on an individual level. Even more so, this would enable monitoring of each participant in the therapy and could enable application adjustment according to the information from these statistical metrics and machine learning techniques. Another type of improvement that can be made in relation to the application is the addition of several different themes to the user interface, which would make it even more customizable to the user. At this point, a completely separate administrator module could be added, through which the parent could make individual settings adjusted to his/her child, but could also enable the parent to follow analyses of the data generated by the application. In the future, machine learning models could be included in the application itself. Another improvement that can be made is to send the data in real-time to a server, which would enable their processing and presentation in real-time. This would allow therapists to remotely monitor children’s progress and intervene appropriately. In support of this, the system itself could include warnings in the case of some unwanted occurrences, such as the reversal of the same action or the subsequent rapid clicking of random places on the screen. Normally, this data transmission should be adequately protected with encryption and, of course, have the prior knowledge and permission of the parents.

## 5. Conclusions

Treatments for children with autism can be difficult in order to keep the child’s attention and make him/her interested. Adding a robot to the therapies facilitates this process and allows the child to maintain attention. It additionally encourages the child to communicate, both with the robot and with the other people around him/her. Applications intended for this type of user, which are also often an integral part of these therapies, behave in a similar way. The interest in technology for children with ASD makes this approach effective in the treatment and opens space for further research and the search for ways to bring the technology closer to this type of user. This would enrich the current treatments, but would also improve their results. The use of such additional tools in therapies opens a considerable space for additional analysis of the data resulting from them. This would mean that in addition to the direct benefit of the tools themselves, with the help of statistical mechanisms and machine learning, useful information can be found that would later contribute to the improvement of the whole process. This generally refers to the detection of patterns of repetitive actions, detection of emotions, speech processing, monitoring of body and eye movements, etc.

The synergy we created between robot treatments and the web/mobile application enabled children to receive therapy virtually even when the therapy involves large hardware requirements and elements that cannot be found at home. This shows that the addition of such an application is successful because it allows children to receive the therapy despite the demands for essential and necessary devices. Enriching robot therapy for children with autism with a complementary application takes the whole approach to a higher level and enables overcoming some of the obstacles that therapists face in this type of therapy. The application created for this research mirrors the therapy process, allowing the users to extend the time of interaction with the robot at home or in any environment outside the therapy room. In such a way, it overcomes a bottleneck in this type of therapy, the lack of therapists and robots. The application mirrors the environment in which the robot is used for the therapy, so the users can easily draw a parallel between the therapy sessions and the scenes and videos used in the application. This similarity allows easy use of the application without the need for prior training, and its modularity allows easy modifications of the scenarios according to the needs of the users. This concept has great potential, and it is certain that its further development and improvement would contribute to even better results in therapies for children with autism.

## Figures and Tables

**Table 1:** Statistics of answers from the questions of the first questionnaire.

Question	Average Answer	Median	Mode
It should be easy to use (for me)	5	5	5
It should be easy to use (for my child)	5	5	5
Navigation and setting should be done in a minimum number of steps	4.9	5	5
I should be able to use it without written instructions	4.85	5	5
My child should be able to use it without written instructions	5	5	5
I should be able to correct wrong clicks quickly and easily (for example, with the “back” button)	5	5	5
I should be able to learn to use it quickly	4.8	5	5
My child should be able to learn to use it quickly	4.9	5	5
The app should have fun or playful elements	4.95	5	5
Video quality is important for my child’s experience	3.85	4	4
The app should have fun or playful elements	4.85	5	5
For my child, the presence of on-screen help messages will be disturbing	3.2	3	3

**Table 2:** Number of answers to the questions of application usefulness questionnaire (after application development).

Question/Answer	1	2	3	4	5
My child enjoys using the app.	3	2	5	5	5
I initiate the use of the application.	0	0	6	3	11
My child initiates the use of the application.	10	2	6	1	1
Meets my needs.	0	1	8	2	9
Meets the needs of my child.	2	0	10	3	5
Does everything I expected.	0	2	4	2	12
Easy to use (for me)	0	0	0	5	15
Easy to use (for my child)	0	1	2	3	14
It takes the least possible steps to achieve what I want to do with the application	0	2	2	1	15
I can use it without written instructions	0	0	1	3	16
I did not notice any inconsistencies while using it	0	1	3	2	14
It is easy to learn to use (for me)	0	0	0	2	18
It is easy to learn to use (for my child)	0	0	4	1	15
Has fun and playful elements	0	0	3	3	14
I’m satisfied with the application	0	0	2	3	15
I would recommend it to a friend.	1	0	4	3	12
The reading of the text on the screen (size, color, font, etc.) is easy	0	0	0	5	15
The visual organization of the information is very clear	0	1	2	1	16
The sequences on the screen are very clear	0	0	3	3	14
The quality of the video is very good	0	1	1	7	11
Exploring new features by trial and error is easy	0	0	3	3	14
Completing tasks is always simple	0	0	2	4	14
For my child, the presence of on-screen help messages is helpful	0	2	3	3	12
For my child, the presence of the on-screen navigation keys is helpful	0	0	6	3	11
The user manual usage is very clear/helpful	1	1	4	1	13
The system speed is fast enough	0	0	2	5	13
The video loading is fast enough	0	0	2	6	12
The system reliability is very good	0	1	3	3	13
The system tends to be silent	0	0	4	2	14
Correcting my mistakes is easy	0	0	5	2	13
Using the system has improved my child’s functioning/behavior/skills	4	3	9	2	2
Using the system has increased the effectiveness of clinical treatment	4	2	9	3	2
I think the system is useful for its intended purpose	1	1	5	1	12

**Table 3:** Categories and number of questions per category.

Category	Number of Questions
Communication skills	9
Shifting gaze and eye contact	4
Reacting to others	10
Turn taking activities	6
Imitation	5
Language skills	9
Vocalization and speech	5
Reason and effect	4
Play	5
Attention	4
Everyday abilities	8
Coping mechanisms	6

**Table 4:** Changes in the answers by patient. Every row represents a questionnaire; columns represent negative, natural, or positive change.

Patient No.	<0	=0	>0
0	9	35	30
1	29	29	16
2	1	18	55
3	5	35	34
4	13	49	12
5	15	34	25
6	21	41	12
7	1	68	5
8	4	48	22
9	2	9	63
10	1	72	1
11	0	2	72
12	1	7	66
13	1	16	57
14	2	59	13
15	2	55	17
16	2	59	13
17	0	41	33
18	1	47	26
19	0	5	69

**Table 5:** Changes in the answers by category. Every row represents a category; columns represent negative, natural, or positive change in percentage.

Category	<0	=0	>0
communication	5	56	39
gaze shift	6	49	45
response	10	44	46
turn taking	11	50	39
imitation	14	38	48
language	1	53	46
vocalization	5	59	36
cause	16	51	33
play	5	52	43
attention	6	43	51
daily	2	40	58
coping	12	56	32

**Table 6:** Categories and number of questions for questionnaire for professionals.

Category	Number of Questions
Frequency of use	2
Total hardware response	13
Total software response	16
Learning to work with the application	3
System capabilities	3
The concept of SMART	4

## Data Availability

Data can be found in the electronic database of the Faculty of Computer Science and Engineering, Ss. Cyril and Methodius University in Skopje, which cannot be publicly disclosed, being subjected to the data protection policy.
